# Demographic profile of HIV and helminth-coinfected adults in KwaZulu-Natal, South Africa

**DOI:** 10.4102/sajid.v38i1.466

**Published:** 2023-01-09

**Authors:** Miranda N. Mpaka-Mbatha, Pragalathan Naidoo, Md. Mazharul Islam, Ravesh Singh, Zilungile L. Mkhize-Kwitshana

**Affiliations:** 1 Department of Biomedical Sciences, Faculty of Natural Sciences, Mangosuthu University of Technology, Umlazi, Durban, South Africa; 2 Department of Medical Microbiology, College of Health Sciences, School of Laboratory Medicine and Medical Sciences, University of KwaZulu-Natal, Durban, South Africa; 3 Division of Research Capacity Development, School of Laboratory Medicine and Medical Sciences, South African Medical Research Council (SAMRC), Cape Town, South Africa; 4 Department of Animal Resources, Ministry of Municipality, Doha, Qatar

**Keywords:** HIV, helminths, neglected tropical diseases, coinfection, demographic profile

## Abstract

**Background:**

Helminth and HIV infections are endemic among poor populations. Studies investigating the socio-demographic and economic risk factors associated with dual HIV and helminth coinfection are scarce.

**Objectives:**

This study aimed to describe risk factors associated with HIV and helminth coinfections among peri-urban South African adults residing in poorly developed areas with high poverty levels, lack of sanitation and a clean water supply.

**Method:**

Adult participants (*n* = 414) were recruited from clinics in the south of Durban, KwaZulu-Natal, South Africa. Participants’ demographic, socio-economic, sanitation and household information, anthropometric measurements and HIV status were collected. Stool samples were donated for coproscopy to detect helminths using the Kato-Katz and Mini Parasep techniques. Blood was collected to confirm participants’ HIV status and to determine *Ascaris lumbricoides*-specific immunoglobulin E (IgE) and immunoglobulin G4 (IgG4) levels to improve microscopy sensitivity.

**Results:**

Overall coinfection was 15%, and single helminth and HIV prevalence were 33% and 52%, respectively. *Ascaris lumbricoides* was predominant (18%). Univariate analysis of variance (ANOVA) showed that coinfection was 11.9% and 19.8%, respectively, among the 18–34 years and 35–59 years age groups (*p* = 0.0006), 16.4% and 19.9%, respectively, for the no income and < R1000.00 groups (*p* = 0.0358) and 22.8% and 17.1%, respectively, for the pit or public toilets and toilets not connected to sewage groups (*p* = 0.0007).

**Conclusion:**

Findings suggest that the dual infection with HIV and helminth infections among adults residing in under-resourced areas with poor sanitary conditions is frequent. Older age, poor toilet use and low income are associated with coinfection. More attention is required to break the cycle of coinfections and possible disease interactions.

**Contribution:**

The study highlights the importance of determining and treating helminth infections among adult population during HIV and helminth coinfection and the influence of poor sanitation and socioeconomic status on disease transmission.

## Introduction

Intestinal helminth parasitic worms are listed by World Health Organization as part of the neglected tropical diseases. They infect over 2 billion (24%) of the world’s population and are endemic in tropical and subtropical climatic regions across East Asia, China, the Americas and sub-Saharan Africa.^1^ The most prevalent intestinal helminth species worldwide are *Ascaris lumbricoides* (1.2 billion), *Trichuris trichiura* (795 million), *Strongyloides stercoralis* (600 million) and *Necator americanus* and *Ancylostoma duodenale* (740 million).^2^ Climate conditions, poverty, unsanitary conditions and a lack of clean water supply promote the transmission, spread and pathogenesis of helminth parasites.^3,4^

As most helminth infections are asymptomatic and associated with lower mortality rates, they have received less attention than more virulent pathogens such as the HIV/AIDS, tuberculosis, malaria^5^ and recently the severe acute respiratory syndrome coronavirus 2 (SARS-CoV-2) virus.^6^ High prevalence levels of helminth infections in children, adults and pregnant women have been reported.^5,7,8^ Helminths are associated with iron deficiency anaemia, intestinal obstruction, malnutrition, malabsorption, retardation of mental and physical growth in children and mortality.^9^

Over 38 million people globally are infected with HIV, the majority of whom are living in low- and middle-income countries with poor socio-economic status.^10^ As both HIV and helminth infections are widespread under similar conditions, the possibility of people being coinfected remains high.^11^ Helminths are known to activate anti-inflammatory T-helper type 2 (Th2) and Th3 regulatory immune responses^12^ while HIV promotes the activation of vital pro-inflammatory Th1/Th17 immune responses. During coinfection, this immunological scenario can be disadvantageous because helminths could possibly dampen the production of vital Th1/Th17 cytokines that are needed to control the early stage of HIV infection.^13^ An association between chronic helminth infections and lower CD4+ counts,^14^ increased HIV viral loads, increased HIV infections^15^ and impaired antiretroviral drug activity has been reported.^16^

In South Africa (SA), the prevalence of HIV and helminth single and coinfection is high, especially among impoverished communities^17,18^ confirming the notion of these being diseases of poverty. KwaZulu-Natal (KZN) province is at the epicentre of HIV globally.^19^ Likewise, because of its subtropical weather, a high prevalence of helminths has been reported in this province.^20^ It is therefore imperative to ascertain whether the known factors of poverty that have been associated with each infection are at play during coinfection, at local contexts. Hence, taking all the above-mentioned facts into consideration, the aim of this study was to describe socio-demographic risk factors associated with HIV and helminth coinfections among peri-urban South African adults residing in underdeveloped areas with high poverty rates, poor sanitary conditions and a lack of clean water supply.^5^

## Methodology

### Study setting

This is a sub-study of a main project that aimed to analyse the immunological and nutritional interactions between HIV and helminth coinfections. The study area is situated in the peri-urban area south of Durban, KZN, SA. It has 11 primary health care clinics (PHC) servicing a total population of 404 811.^21^ Of the 11 clinics, five were selected as study sites based on the availability of HIV counselling and testing (HCT) services. The study was conducted between March 2020 and May 2021.

### Participant recruitment and selection

Participants were recruited from the selected PHC clinics. The study participants were adults (≥ 18 years) who were attending the clinics. Some participants were accompanying patients to the clinic; others were community people who were recruited by family members who attended the clinics as well as clinic personnel working in each of the selected clinics. In the clinic waiting area, an information session was conducted to inform the participants about the objectives, possible outcomes and benefits of the study. Those who were willing to participate in the study were provided with further information individually, such as their rights and responsibilities during the study. A bilingual informed consent form for study participation was prepared and administered in the English and the local isiZulu languages. Participants were taken through the consent forms and were included in the study once they had voluntarily signed the consent form. Confidentiality was maintained by protecting all personal data that may identify the study participants through the use of study identification numbers.

### Data collection

Pre-screening was carried out to identify those who were ineligible if they were (1) on chronic medication for diseases such as diabetes mellitus, cancer, cardiovascular diseases, (2) abusing alcohol (more than two drinks daily) or illicit drugs, (3) pregnant, (4) frail and very sick and (5) mentally challenged and unable to give informed consent. Eligible participant’s weight and height were recorded. Participants’ body mass index (BMI) were classified as underweight (< 18.5 kg/m^2^), normal weight (18.5 kg/m^2^ – 24.9 kg/m^2^), overweight (25.0 kg/m^2^ – 29.9 kg/m^2^) and obese (≥ 30 kg/m^2^).^5^

A questionnaire was used to collect the participants demographic information, including history of worm infection, household information, income level, toilet use and water source.

### Sample collection and laboratory analysis

The participants were asked to donate two stool samples for parasite detection, over a 2-day period to increase the sensitivity and specificity of coproscopy. Whole blood was collected from all participants using the Vacuette^®^ ethylenediaminetetraacetic acid (EDTA) and serum separator tubes (SST). Upon arrival of the stool samples in the laboratory, they were processed using the Kato-Katz technique and the modified faecal parasite concentrator technique Mini Parasep^®^ SF followed by coproscopy for helminth parasite detection (eggs and worms).^22^ Blood was used for the Alere DetermineTM HIV-1/2 Ag/Ab Combo rapid test (Orgenics Ltd, Israel), which was used to confirm the participant’s HIV status. Inconclusive results were confirmed by using the ICT HIV-1/2 Ag/Ab test kit (ICT International). *Ascaris lumbricoides*-specific IgE and IgG4 was analysed using Phadia™ 200 instrument (ThermoFisher Scientific).

### Statistical analysis

The participants demographic and laboratory data were entered into a Microsoft Excel 2016 spreadsheet, and statistical analyses were performed using Stata/IC- 17 (Stata Corp LLC, Lakeway Drive, TX, United States [US]). Descriptive analysis was performed to determine the overall prevalence 95% confidence interval. Univariate analysis was carried out by the one-way analysis of variance (ANOVA) Bartlett’s equal-variance test in identifying the association between HIV, helminth and HIV-helminth coinfection and different demographic factors. The results have been presented as the total positive (*n*), percentage (%) and *p*-value (*p*-value < 0.05 was considered statistically significant).

### Ethical considerations

This study was undertaken following the approval by the Biomedical Research Ethics Committee (BREC) at the University of KwaZulu-Natal for both the main study (BE351/19) and the substudy (BREC/00001495/2020). Throughout the study, ethical principles for research on human participants were followed, including autonomy, justice, beneficence, nonmaleficence, confidentiality and dignity.

## Results

A total of 414 participants were recruited, of those 128 (30.9%) were uninfected and 214 (52%) were infected with HIV; 137 (33%) infected with helminths and 62 (15%) dually infected with both HIV and helminths.

### Characteristics of study participants

The demographic profiles of study participants are shown in Table 1. The majority of participants were single (70%), 35–59 years of age (46%), females (67%), overweight and obese (60%) and unemployed (78%). Twenty-one percent had primary school education or below while the majority (68%) had secondary school education. Regarding earnings, 22% were employed and had a stable salary. For the remainder, 13% had no income, the majority (45%) were dependent on the state social support (16% pension and 29% social grant). Furthermore, income analysis showed that 85% earned less than R5000.00 (< R1000.00 [44%] and > R1000.00 – ≤ R5000.00 [41%]). These groups constitute 78% of the study population being unemployed or falling within the low-income bracket. A proportion (20%) lived in informal houses or houses made up of mud and stones and 30% lived in houses with < 2 rooms. The probability of having 5–8 and > 8 family members living in a home was 43% and 8%, respectively. Over 40% of participants had one or more children attending preschool while 63% had one or more children attending primary school in the home. With regard to toilet facilities, 33% used pit toilets and 8% used toilets with the flush system not connected to the sewage. With regard to water source, 44% relied on taps located outside the house, while 16% relied on public taps. A further 17% relied on unclean water sources. Approximately, 26% of participants had a history of previous helminth infection.

**TABLE 1:** Demographic profiles of study participants.

Participant characteristics	*n*	%	95% confidence interval
**Age (years)**			
18–34	160	38.7	33.9–4.53
35–59	212	51.2	46.3–56.1
≥ 60	42	10.1	7.4–13.7
**Gender**			
Male	138	33.3	29.0–38.0
Female	276	67.0	62.0–7.0
**Body mass index (kg/m^2^)**			
< 18.5	24	5.8	3.9–8.5
18.5–24.9	138	33.3	2.9–3.8
25.0–29.9	111	26.8	22.8–31.3
≥ 30	141	34.1	30.0–39.0
**Marital status**			
Married	59	14.3	11.2–18.0
Single	291	70.3	65.7–74.5
Divorced/widowed	22	5.3	3.5–7.9
Living together	42	10.1	8.0–13.4
**Education level**			
Primary school or below	85	20.5	16.9–25.0
Secondary	281	68.0	63.2–72.2
Tertiary	48	12.0	9.0–15.0
**Employment status**			
Employed	90	21.7	18.0–26.0
Unemployed	324	78.3	74.0–82.0
**Income source**			
Salary	90	21.7	18.0–26.0
No income	54	13.0	10.1–16.6
Pension	64	15.5	12.3–19.3
Dependant	48	11.6	9.0–15.0
Part time job	39	9.4	7.0–12.6
Social grant support	119	28.7	25.0–33.3
**Income level**			
No income	55	13.3	10.4–17.0
< R1000.00	181	43.7	39.0–49.0
> R1000.00 – ≤ R5000.00	171	41.3	37.0–46.1
> R5000.00	7	2.0	0.8–3.5
**Toilet use**			
Pit	136	33.0	28.5–37.5
Flush connected to sewage	243	58.7	54.0–63.3
Flush not connected to sewage	35	8.5	6.1–11.5
**House type**			
Brick/block	329	79.5	75.3–83.1
Informal houses	66	16.0	12.7–20.0
Mud and/or stone houses	19	4.6	3.0–7.0
**Number of rooms in house**			
1	63	15.2	12.1–18.9
2	64	15.5	12.3–19.3
3	40	10.0	7.2–13.0
> 3	247	60.0	54.9–64.3
**Number of people at home**			
< 5	203	49.0	44.3–53.8
5–8	176	42.5	37.8–47.3
> 8	35	8.5	6.1–11.5
**Pre-school children at home**			
0	247	59.7	54.9–64.3
1	109	26.3	22.3–30.8
> 1	58	14.0	10.9–18.0
**Primary school children at home**			
0	152	36.7	32.2–41.5
1	127	31.0	26.4–35.3
> 1	135	32.3	28.3–37.3
**Source of water**			
Tap inside house	145	35.0	30.6–39.7
Tap outside house	184	44.4	37.7–49.3
Public tap	68	16.4	13.2–20.3
Others	17	4.1	2.6–6.5
**Previous helminth infection**			
Yes	107	25.8	22.0–30.3
No	307	74.2	69.7–78.1

R, South African rand.

### Distribution of helminth species in the study population

**FIGURE 1:**
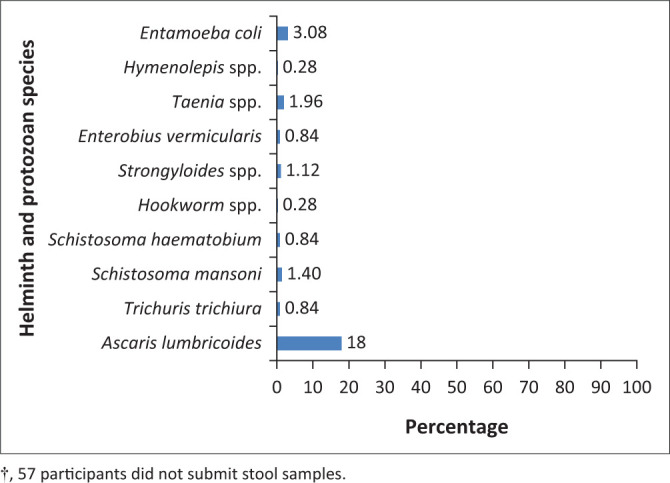
Helminth and *Entamoeba coli* prevalence by species in the study population (*n* = 357†).

The distribution of the various helminth and protozoa species in the study population is highlighted in Figure 1. Of the total study population (*n* = 414), 33% (*n* = 137) were infected with helminths and 3.1% (*n* = 11) were infected with *Entamoeba coli* (based on stool coproscopy and *A. lumbricoides*-specific IgE and IgG4 results). The species detected in the participants who were diagnosed with helminthiasis by eggs or ova in their stool samples (*n* = 357) were *A. lumbricoides* (18%), *Taenia* spp. (1.96%), *Schistosoma mansoni* (1.40%), *Strongyloides* spp. (1.12%), *T. trichiura* (0.84%), *Schistosoma haematobium* (0.84%), *Enterobius vermicularis* (0.84%), *hookworm* spp. (0.28%) and *Hymenolepis* spp. (0.28%). *E. coli* (protozoan) (3.08%) were also prevalent (Figure 1).

### Association between coinfection status and demographic variables

The association between HIV and helminth coinfection and demographics variables are presented in Table 2. Significance was noted for age (*p* < 0.0001) ), marital status (*p* = 0.023), education level (*p* = 0.004), source of income (*p* = 0.015), income level (*p* = 0.036), number of primary school children residing in the home (*p* = 0.008) and toilet use (*p* = 0.015). In the age group 18-34 years, 11.9% were coinfected, while 19.8% were coinfected in age group 35–59 years. Income level < R1000.00 (20%) were coinfected, while 43.1% were only HIV infected and 13.3% were only helminth infected. Toilet use coinfection among those who use pit or public toilets and toilets not connected to sewerage were 17% – 23%, while in the helminth only infected group were 16% – 23% (Table 2).

**TABLE 2:** Univariate association of HIV, helminth, and HIV-helminth co-infection with different demographic characteristics.

Patient characteristics	*N*	Negative for both HIV and helminth		HIV-infected		Helminth-infected		HIV and helminth co-infected		*p*
		*n*	%	*n*	%	*n*	%	*n*	%	
**Total**	414	128	30.9	152	36.7	72	17.4	62	15.0	-
**Age**										**< 0.0001**
18–34	160	56	35.0	51	31.9	34	21.3	19	11.9	
35–59	212	46	22.0	96	45.3	28	13.2	42	19.8	
≥ 60	42	26	61.9	5	11.9	10	23.8	1	2.40	
**Gender**										0.324
Male	138	44	32.0	46	33.3	30	21.7	18	13.0	
Female	276	84	30.4	106	38.4	42	15.2	44	25.0	
**Body Mass Index**										0.144
Under weight	24	5	20.8	12	50.0	5	20.8	2	8.3.0	
Normal	138	35	25.4	41	29.7	25	18.0	29	21.0	
Overweight	111	38	34.2	49	44.1	20	18.0	12	10.8	
Obese	141	50	35.5	50	35.5	22	15.6	19	13.5	
**Marital status**										**0.023**
Single	291	85	29.2	107	36.8	51	17.5	48	16.5	
Married	59	27	46.0	14	23.7	13	22.0	7	12.0	
Living together	42	7	17.0	24	57.1	4	9.5	6	14.3	
Divorced and Window	22	9	41.0	7	31.8	5	22.7	2	9.1	
**Education level**										**0.004**
Primary or below	85	37	43.5	20	23.5	21	24.7	7	8.2	
Secondary	281	76	27.0	113	40.2	42	14.9	50	18.0	
Tertiary	48	15	31.3	19	39.6	9	18.8	5	10.4	
**Employment status**										0.065
Employed	90	25	28.0	39	43.3	15	16.7	11	12.2	
Unemployed	324	103	32.0	113	34.9	58	17.9	51	15.7	
**Source of income**										**0.015**
Salary	90	25	28.0	40	44.4	15	16.7	11	12.2	
No Income	54	17	31.5	14	25.9	14	25.9	9	17.0	
Pension	64	31	48.4	16	25.0	13	20.3	5	7.8	
Dependent	48	11	23.0	26	54.2	7	14.6	5	10.4	
Social support grant	119	34	29.0	47	39.5	15	12.6	23	19.3	
Part-time job	39	10	25.6	12	31.0	8	20.5	9	23.1	
**Income level**										**0.007**
No income	55	17	30.9	14	25.5	15	27.3	9	16.4	
< R1000.00	181	43	24.0	78	43.1	24	13.3	36	20	
> R1000.00 to ≤ R5000.00	171	66	39.0	59	34.5	32	18.7	17	9.9	
> R5000.00	7	2	29.0	1	14.3	1	14.3	0	0.0	
**House type**										0.453
Brick/block	329	107	32.5	117	35.6	60	18.2	44	13.4	
Shacks	66	17	26.0	27	40.9	9	13.6	13	19.7	
Mud/ stone houses	19	4	21.1	7	36.8	3	15.8	5	26.3	
**Number of rooms in house**										0.378
1	63	17	27.0	28	44.4	8	12.7	9	14.3	
2	64	25	39.1	20	31.3	7	10.9	12	19.0	
3	40	11	3.0	16	40.0	10	25.0	3	7.50	
> 3	247	75	30.4	88	35.6	46	18.6	38	15.4	
**Toilet use**										**0.015**
Pit and public	136	33	24.3	48	35.3	23	16.9	31	23.0	
Sewage connected	243	88	36.1	91	37.4	38	15.6	25	10.3	
Sewage not connected	35	7	20.0	13	37.1	8	22.9	6	17.1	
**Source of water**										0.206
Tap inside house	145	47	32.4	42	29.0	20	13.8	21	14.5	
Tap outside house	184	61	33.2	78	42.4	34	18.5	25	14.0	
Public tap	68	15	22.1	28	41.2	15	22.1	10	14.7	
Others†	17	5	29.4	4	23.5	2	11.8	6	35.3	
**Total people at home**										0.612
< 5	203	62	30.5	75	36.9	40	19.7	26	12.8	
5–8	176	56	31.8	61	34.7	27	15.3	32	18.2	
> 8	35	10	29.0	16	45.7	5	14.3	4	11.4	
**No. of pre-school children**										0.061
0	247	69	27.9	98	39.7	42	17.0	38	15.4	
1	109	41	37.6	29	26.6	18	16.5	21	19.3	
> 1	58	18	31.0	25	43.1	12	20.7	3	5.2	
**No. of primary school children**										**0.008**
0	152	46	30.1	52	35.2	33	21.7	21	13.8	
1	127	49	39.0	45	35.4	15	11.8	18	14.2	
> 1	135	33	24.4	55	40.7	24	17.8	23	17.0	

Note: Univariate ANOVA analysis showed that older age (35-59), poor toilet use (pit and not connected to sewerage) and low income, source of income, marital status, education level, number of primary school children residing in the home were associated with HIV and Helminth coinfection. Data in Table 2 were analysed using the Chi-squared test. A Chi-squared *p* < 0.05 was considered statistically significant (*p*-values in bold).

*R*, South African rand.

† , Water from the following sources: rivers, boreholes and municipal water delivery trucks.

## Discussion

This study investigated the socio-demographic and economic risk factors associated with HIV and helminth coinfections among South African adults residing in poorly resourced areas in the eThekwini south region that has high poverty rates, poor sanitary conditions and a lack of clean water supply. Findings suggest that older age, low-income level and poor toilet use are the main factors associated with helminth and HIV co-infection in this study population. The HIV and helminth coinfections (19.8%) were most common in people aged 35–59 years, and HIV single infection was 45.3% in the same age group. Those between the age group 18–34 years had HIV and helminth coinfection prevalence of 11.9% and HIV-single infections recorded at 31.9%. The findings of this study are consistent with the HIV national norms, which show that there are approximately 8.2 million people living with HIV in SA between the ages of 15–49 years, accounting for at least 19.5% of the HIV-infected population.^23^ The overall HIV prevalence in this study population was 52%, which is consistent with the fact that participants were recruited from HCT clinics.

Fifteen per cent of this adult population is coinfected with HIV and helminths. Only a few South African studies^5,14,17^ have reported on this potentially debilitating dual burden, particularly among adults where peak HIV infections occur. The HIV and helminth coinfection was 15.7% in the unemployed group, while the group had a higher occurrence of HIV and helminth single infections, which were 51% and 33%, respectively. The HIV and helminth are both poverty-related illnesses; hence these findings were expected.^24^ Following the Eastern Cape, KZN is one of the poorest provinces in SA.^14,25^ Despite the fact that the majority (68%) of the study participants had secondary school education, 78% are unemployed and many depend on government grants and family support for income. This may be attributed to the high unemployment rate in SA^26^ and KZN, worsening the burden and impact of HIV and helminth infections and diseases that are being superimposed upon poverty-stricken individuals. Related to this, those who use pit or public toilets had a higher occurrence of HIV and helminth coinfections (22%) and HIV, helminth single infections (58%) and (40%), respectively.

The overall prevalence of intestinal helminth parasites was 33%, which was lower than 38% that was reported in a similar population of HIV-infected adults from the eThekwini North district by Mkhize et al.^5^ In this study, *A. lumbricoides* (18%) was the most prevalent helminth infection followed by Schistosoma infections (1.68%), contrary to previously reported studies in which *T. trichiura* was the second highest after *A. lumbricoides* in SA.^25,27^ Preschool and primary school children are the most vulnerable to helminth infections and are the most likely to transfer the infection to their adult family members.^28^ As such, in KZN, several studies have reported a high prevalence of these infections that ranged between 20% and 80% among children over several decades.^24,25,29^

The HIV and helminth infections are most common in KZN particularly in areas that are poverty stricken, with inadequate sanitation,^5,25^ as well as those who live in small, overcrowded houses.^28^ Furthermore, HIV infection is reported to be more common among females.^26^ Several reasons have been suggested that relate to female predilections including the anatomical structure of women reproductive system, which predisposes women to be more vulnerable to sexually transmitted infections. Another factor is health-seeking behaviours, which vary between men and women.^28^ The latter is also demonstrated in this study, which has predominantly female (66%) participants.

Transmission of helminth infections is exacerbated in communities that rely on pit toilets and toilets not connected to sewerage systems^5,30^ and areas that highly depend on outdoor or public taps as a source of water for daily consumption and sanitary use.^30^ The findings of this study concur with these reports. Although the helminth re-infection rate was not analysed in this study; other studies reported a high helminth re-infection rate among people who were continuously being exposed to poor socioeconomic and unsanitary lifestyles after deworming treatment.^31^ This finding emphasises the importance of good hygiene practices and the need for the local government to enhance sanitation in endemic areas to reduce and break the helminth transmission cycle.

This study’s outcomes justify the need to include adults in deworming programmes because such initiatives are only targeted to school-going children in most cases.^32,33^ Furthermore, preventative chemotherapy is especially crucial for HIV-infected adults because chronic helminth infections are suggested to have a deleterious influence on the immune system even though the results of these studies are limited.^12,34,35^

The study is limited by selection bias because most of the participants were recruited from clinics, even though some of the controls were recruited from the community and people accompanying the patients. In particular, the selection of clinics was biased towards those with HCT clinics. This is reflected in the high overall prevalence of HIV in the study sample.

## Conclusion

Although the associations between poverty and HIV and helminths as single infections have been reported,^14,36^ the association during dual or coinfection has not been investigated in the local setting of SA. In the main, a similar pattern of an association with poverty indicators has been found in this study, thus adding to the existing knowledge base. Mainly low income, poor toilet usage and older age were associated with coinfection.

These findings highlight the need to control the transmission of HIV and helminth infections among adults residing in resource-limited areas with poor sanitary conditions. Relevant stakeholders need to develop health intervention programmes including infrastructure for proper sanitation and clean water supply. Education of the affected groups on the importance of proper hygiene practices, adherence to antiretroviral therapy and deworming chemotherapy to control HIV and helminth infections would be the most feasible while other costly interventions are being planned. Finally, the impact of coinfection on the host immune response and how this affects the efficacy of antiretroviral drug therapy, the administration of deworming chemotherapeutic programmes in the coinfected groups are all largely under-researched.

## References

[1] World Health Organization. Soil-transmitted helminth infections [homepage on the Internet]. 2022 [cited 2022 Jun 13], p. 502–527. Available from: https://www.who.int/news-room/fact-sheets/detail/soil-transmitted-helminth-infections

[2] Centers for Disease Control and Prevention. Parasites – Soil-transmitted helminths [homepage on the Internet]. 2021 [cited 2022 Jun 13]. Available from: https://www.cdc.gov/parasites/sth/index.html

[3] Çeliksöz A, Güler N, Güler G, Öztop AY, Degerli S. Prevalence of intestinal parasites in three socioeconomically-different regions of Sivas, Turkey. J Health Popul Nutr [serial online]. 2005 [cited 2022 Jun 23];23(2):184–191. Available from: https://pubmed.ncbi.nlm.nih.gov/16117371/16117371

[4] Woh PY, Lin Thong K, Behnke JM, Lewis JW, Nursheena S, Zain M. Evaluation of basic knowledge on food safety and food handling practices amongst migrant food handlers in Peninsular Malaysia. Food Control. 2016;70:64–73. 10.1016/j.foodcont.2016.05.033

[5] Mkhize BT, Mabaso M, Mamba T, Napier CE, Mkhize-Kwitshana ZL. The interaction between HIV and intestinal helminth parasites coinfection with nutrition among adults in KwaZulu-Natal, South Africa. Biomed Res Int. 2017;2017:1–12. 10.1155/2017/9059523 PMC5380830 28421202

[6] Naidoo P, Ghazi T, Chuturgoon AA, et al. SARS-CoV-2 and helminth co-infections, and environmental pollution exposure: An epidemiological and immunological perspective. Environ Int. 2021;156:106695. 10.1016/j.envint.2021.106695 34171587 PMC8205275

[7] Harhay MO, Horton J, Olliaro PL. Epidemiology and control of human gastrointestinal parasites in children. Expert Rev Anti Infect Ther. 2010;8(2):219–234. 10.1586/eri.09.119 20109051 PMC2851163

[8] Adegnika AA, Agnandji ST, Chai SK, et al. Increased prevalence of intestinal helminth infection during pregnancy in a Sub-Saharan African community. Wien Klin Wochenschr. 2007;119(23–24):712–716. 10.1007/s00508-007-0907-z 18157604

[9] Allen JE, Maizels RM. Immunology of human helminth infection. Int Arch Allergy Immunol. 1996;109(1):3–10. 10.1159/000237225 8527948

[10] World Health Organization. Sheets/Detail/HIV/AIDS WN 30 N 2021. HIV/AIDS [homepage on the Internet]. 2021 [cited 2022 Jan 23]. Available from: https://www.who.int/news-room/fact-sheets/detail/hiv-aids

[11] Oliveira SC, Figueiredo BC, Cardoso LS, Carvalho EM. A double edged sword: Schistosoma mansoni Sm29 regulates both Th1 and Th2 responses in inflammatory mucosal diseases. Mucosal Immunol. 2016;9(6):1366–1371. 10.1038/mi.2016.69 27554296

[12] Motran CC, Silvane L, Chiapello LS, et al. Helminth infections: Recognition and modulation of the immune response by innate immune cells. Front Immunol. 2018;9(APR):664. 10.3389/fimmu.2018.00664 29670630 PMC5893867

[13] Borkow G, Bentwich Z. Chronic immune activation associated with chronic helminthic and human immunodeficiency virus infections: Role of hyporesponsiveness and anergy. Clin Microbiol Rev. 2004 Oct;17(4):1012–1030. 10.1128/CMR.17.4.1012-1030.2004 15489359 PMC523563

[14] Adeleke OA, Yogeswaran P, Wright G. Intestinal helminth infections amongst HIV-infected adults in Mthatha General Hospital, South Africa. Afr J Prim Health Care Fam Med. 2015;7(1):910. 10.4102/phcfm.v7i1.910 26842519 PMC4729221

[15] Elliott AM, Mawa PA, Joseph S, et al. Associations between helminth infection and CD4+ T cell count, viral load and cytokine responses in HIV-1-infected Ugandan adults. Trans R Soc Trop Med Hyg. 2003;97(1):103–108. 10.1016/S0035-9203(03)90040-X 12886815

[16] Mkhize-Kwitshana ZL, Tadokera R, Mabaso MHL. Helminthiasis: A systematic review of the immune interactions present in individuals coinfected with HIV and/or tuberculosis. In: Rodrigo L, editor. Human helminthiasis [homepage on the Internet]. IntechOpen; 2017 [cited 2022 Jun 27]. Available from: https://www.intechopen.com/chapters/53206

[17] Mkhize-Kwitshana ZL, Mabaso MLH, Walzl G. Proliferative capacity and cytokine production by cells of HIV-infected and uninfected adults with different helminth infection phenotypes in South Africa. BMC Infect Dis. 2014 Dec 11;14(1):499. 10.1186/1471-2334-14-499 25209883 PMC4262143

[18] Fincham JE, Markus MB, Adams VJ. Could control of soil-transmitted helminthic infection influence the HIV/AIDS pandemic. Acta Trop. 2003 May 1;86(2–3):315–333. 10.1016/S0001-706X(03)00063-9 12745148

[19] Allinder S. The world’s largest HIV epidemic in crisis: HIV in South Africa [homepage on the Internet]. Center for Strategic and International Studies. Centre for Strategic and International Studies; 2019 [cited 2022 Jun 28]. Available from: https://www.csis.org/analysis/worlds-largest-hiv-epidemic-crisis-hiv-south-africa

[20] Mabaso MLH, Appleton CC, Hughes JC, Gouws E. The effect of soil type and climate on hookworm (Necator americanus) distribution in KwaZulu-Natal, South Africa. Trop Med Int Heal. 2003;8(8):722–727. 10.1046/j.1365-3156.2003.01086.x 12869093

[21] Statistics South Africa. Province 5 census 2011 - KwaZulu-Natal [homepage on the Internet]. 2011 [cited 2022 Nov 08]. Available from: https://census2011.adrianfrith.com/place/5

[22] Adugna S, Kebede T, Mekonnen Z, Degarege A, Liang S, Erko B. Diagnostic performance of Mini Parasep^®^ solvent-free faecal parasiteconcentrator relative to Kato-Katz and McMaster for the diagnosis of intestinal parasiticinfections. Trans R Soc Trop Med Hyg. 2017;111(12):572. 10.1093/trstmh/try010 29509952 PMC6543883

[23] Stats SA. Statistical release P0302 2000 [homepage on the Internet]. 2021 [cited 2022 Feb 14]. Available from: www.statssa.gov.za

[24] Sacolo-Gwebu H, Chimbari M, Kalinda C. Prevalence and risk factors of schistosomiasis and soil-transmitted helminthiases among preschool aged children (1–5 years) in rural KwaZulu-Natal, South Africa: A cross-sectional study. Infect Dis Poverty. 2019;8(1):47. 10.1186/s40249-019-0561-5 31202273 PMC6571117

[25] Zulu SG, Kjetland EF, Gundersen SG, Taylor M, Zulu S. Prevalence and intensity of neglected tropical diseases (schistosomiasis and soil-transmitted helminths) amongst rural female pupils in Ugu district, KwaZulu-Natal, South Africa. S Afr J Infect Dis. 2020;35(1):123. 10.4102/sajid.v35i1.123 34485471 PMC8377948

[26] Stats SA. Statistical release P0211 [homepage on the Internet]. [cited 2022 Jun 21]; Available from: www.statssa.gov.za

[27] Müller I, Yap P, Steinmann P, et al. Intestinal parasites, growth and physical fitness of schoolchildren in poor neighbourhoods of Port Elizabeth, South Africa: A cross-sectional survey. Parasit Vectors. 2016;9(1):1–13. 10.1186/s13071-016-1761-5 27595566 PMC5011914

[28] Currie DH, Wiesenberg SE. Promoting women’s health-seeking behavior: Research and the empowerment of women. Health Care Women Int. 2003 Dec;24(10):880–899. 10.1080/07399330390244257 14742127

[29] Schutte CHJ, Eriksson IM, Anderson CB, Lamprecht T. Intestinal parasitic infections in Black scholars in northern KwaZulu. S Afr Med J [serial online]. 1981 [cited 2022 Jul 1];60(4):137–141. Available from: https://pubmed.ncbi.nlm.nih.gov/7256455/7256455

[30] Younes N, Behnke JM, Ismail A, Abu-Madi MA. Socio-demographic influences on the prevalence of intestinal parasitic infections among workers in Qatar. Parasit Vectors. 2021;14(1):1–13. 10.1186/s13071-020-04449-9 33472686 PMC7816503

[31] Hesham Al-Mekhlafi M, Surin J, Atiya AS, Ariffin WA, Mohammed Mahdy AK, Che Abdullah H. Pattern and predictors of soil-transmitted helminth reinfection among aboriginal schoolchildren in rural Peninsular Malaysia. Acta Trop. 2008;107(2):200–204. 10.1016/j.actatropica.2008.05.022 18582430

[32] World Health Organization. Preventive in human helminthiasis chemotherapy [homepage on the Internet]. 2006 [cited 2022 Nov 15]. Available from: https://apps.who.int/iris/bitstream/handle/10665/43545/9241547103_eng.pdf?sequence=1

[33] World Health Organization. WHO recommends large-scale deworming to improve children’s health and nutrition [homepage on the Internet]. 2017 [cited 2022 Jun 24]. Available from: https://www.who.int/news/item/29-09-2017-who-recommends-large-scale-deworming-to-improve-children-s-health-and-nutrition

[34] Salgame P, Yap GS, Gause WC. Effect of helminth-induced immunity on infections with microbial pathogens. Nat Immunol. 2013;14(11):1118. 10.1038/ni.2736 24145791 PMC4955540

[35] McSorley HJ, Maizels RM. Helminth infections and host immune regulation. Clin Microbiol Rev. 2012;25(4):585. 10.1128/CMR.05040-11 23034321 PMC3485755

[36] Gall S, Müller I, Walter C, et al. Associations between selective attention and soil-transmitted helminth infections, socioeconomic status, and physical fitness in disadvantaged children in Port Elizabeth, South Africa: An observational study. PLoS Negl Trop Dis. 2017;11(5):e0005573. 10.1371/journal.pntd.0005573 28481890 PMC5436891

